# Organoids in virology

**DOI:** 10.1038/s44298-024-00017-5

**Published:** 2024-02-07

**Authors:** Julie T. S. Chu, Mart M. Lamers

**Affiliations:** Programme in Emerging Infectious Diseases, Duke-NUS Medical School, Singapore, Singapore

**Keywords:** Virology, Immunopathogenesis

## Abstract

To adequately prepare against imminent disease outbreaks from diverse and ever-changing viral pathogens, improved experimental models that can accurately recapitulate host-virus responses and disease pathogenesis in human are essential. Organoid platforms have emerged in recent years as amenable in vitro tools that can bridge the limitations of traditional 2D cell lines and animal models for viral disease research. We highlight in this review the key insights that have contributed by organoid models to virus research, the limitations that exist in current platforms, and outline novel approaches that are being applied to address these shortcomings.

## Introduction

Emerging zoonotic viruses have had a profound impact on the early twenty-first century. Influenza virus, Ebola virus, Zika virus, and the pandemic severe acute respiratory syndrome coronavirus 2 (SARS-CoV-2) have collectively exemplified the substantiality of public health threat posed by emerging viruses. At the same time, urbanization^1^, climate change^2^ and wildlife trade^3^ are predicted to increase the propensity for viral disease emergence in the coming decades. As we usher in a new era of infectious disease, novel approaches to establish greater outbreak preparedness need to be employed. Within this framework, development of physiologically relevant laboratory models for studying disease pathophysiology and development of therapeutics to relieve disease burden needs to be prioritized.

In recent years, organoids have gained significant recognition as physiologically relevant in vitro platforms in the realm of virus research, and existing platforms are being refined continuously to better accommodate human viral disease studies. In this review, we will highlight key virological findings that have been uncovered using organoid models. Additionally, we will discuss the limitations of existing organoid platforms, particularly with relevance to virological research, and novel strategies that are being explored to overcome these shortcomings.

### Traditional experimental models for viral diseases

Two-dimensional (2D) cultured cell lines have served as tools for virus propagation since the early 1900s^4^, and remain the most used platform for studying viral diseases. Traditional 2D cell lines are easy to handle, affordable to maintain and amenable to a wide range of experimental techniques. However, most cell lines are genetically immortalized, cancerous, or transformed to enable long-term culturing, thus often possess defects such as dysfunctional innate immune signaling pathways. Inherent differences between 2D cell lines and normal cells in vivo can result in bottlenecks that select for cell line adapted viral mutants with altered entry receptor usage^5^, transmissibility^6^ and pathogenicity^7,8^ compared to their clinically isolated forms. An alternative in vitro platform is primary cells derived directly from patient tissues, which are of greater resemblance to human cells in vivo, but rely on steady sources of fresh tissue to generate as they typically can only be passaged a few times before showing signs of senescence^9^.

Animal models are used to recapitulate viral disease processes that occur at the whole organism level. Model organisms have served as valuable platforms for determining disease-causative viral agents in fulfillment of Koch’s postulates^10,11^ and as preclinical platforms for vaccines and antivirals^12,13^. Certain physiological traits encompassed by individual animal models that are of high resemblance to those in humans have also been harnessed to study viral disease pathogenesis^14^. For example, ferrets are a favorable model for studying transmission of influenza viruses, owing to the high histological and anatomical similarity of their respiratory tracts to those of human^15^. However, inherent differences between host species limit clinical translatability of findings from animal studies, since viral disease is the end product of a myriad of virus-host interactions that are fine-tuned by evolution. Moreover, as viruses generally exhibit a narrow host range^16^, studies of human viruses in animal models often require extensive rounds of virus adaptation in the target species, resulting in adapted variants with mutations that may alter key viral phenotypes that are relevant to human disease^17–19^. Alternatively, specialized transgenic or humanized organisms with engineered susceptibility towards the viral target may be employed, but are generally costly, time consuming and labor intensive to generate^20–22^.

Given the shortcomings of traditional experimental platforms in authentically recapitulating viral disease in human, developing improved models that can better represent cellular phenotype diversity, disease susceptibility and host responses, whilst being amenable to a wide range of experimental techniques, is a priority in virology. In the past decade, human stem cell-derived organoids have proven repeatedly to be an asset to virus research, unraveling critical viral disease mechanisms that were previously undeterminable in traditional experimental platforms, identifying viral and host factors that facilitate infection, and providing tools for propagating viruses that were previously challenging to cultivate in vitro. Applicability of organoid platforms to virus research was further demonstrated when they were rapidly employed for disease modeling early during the COVID-19 pandemic and have since contributed a wealth of knowledge towards the disease. In this review, we discuss how organoids have been employed to fulfill critical knowledge gaps in virology. We focus our discussion on viruses that have been studied extensively using organoid models in recent years.

### Organoid models for viral diseases

**Fig. 1:**
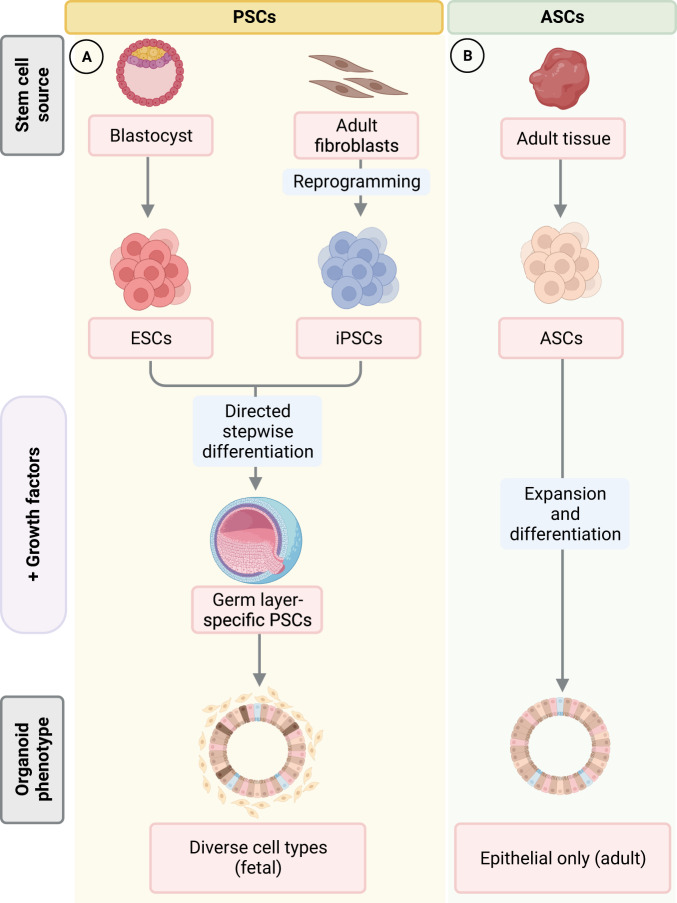
Generation of adult (ASC) and pluripotent stem cell (PSC)-derived organoid models. **A** Embryonic (ESC) or induced pluripotent stem cells (iPSCs), together referred to as PSCs, can be derived from embryonic tissues or reprogrammed from terminally differentiated fibroblasts (and other somatic cells), respectively. These progenitors can be induced to form PSC organoids from all three germ layers through a stepwise differentiation process that requires supplementation of specific growth factors at each stage. PSC organoids contain niche components from the stroma in addition to epithelial cell types, but often present fetal or neonatal phenotypes that closer resemble developing organs rather than adult tissues. **B** ASC organoids are generated from stem cells isolated from adult tissues that have regenerative potential. Following isolation and expansion, ASCs can be cultured in vitro with specific combinations of growth factors to induce formation of organoids containing diverse epithelial cell types from the tissue origin. Figure created with BioRender.

Organoids are defined as multicellular structures or “mini-organs” derived from stem or progenitor cells that are composed of organ-specific cell types with the capacity to self-organize through cell sorting and spatially restricted lineage commitment^23,24^. Organoid models are generally three-dimensional (3D) structures that are either embedded in an extracellular matrix (ECM) gel or free-floating in medium, and require exogenous supplementation of growth factors that are essential for stem cell proliferation. In recent years, many 2D organoid models composed of cells that are directly grown on a flat surface with or without ECM coating, have also been established^25–28^. Organoids can either be derived from embryonic or induced pluripotent stem cells (together referred to as PSCs) or adult stem cells (ASCs) (Fig. 1). PSC-derived organoids from fetal tissues or somatic cells are generated through a stepwise differentiation process that requires supplementation of specific growth factors at each stage^29,30^. Additionally, PSCs can be induced to differentiate into a wide range of tissue types from all three germ layers. Since PSC organoids are formed through processes that are unique to embryonic development, they make for ideal models to study organogenesis, in vivo development, and viral diseases known to impact these processes. ASC-derived organoids, which were first established for the intestine after the identification of Lgr5 as a marker of adult gut stem cells^31,32^, can be generated from tissue-resident stem cells in adult tissues or organs through mimicking the adult stem cell niche during tissue renewal or damage repair. Resultant ASC-derived organoids model adult cells in vivo with high accuracy, but generally only contain epithelial cells.

**Fig. 2:**
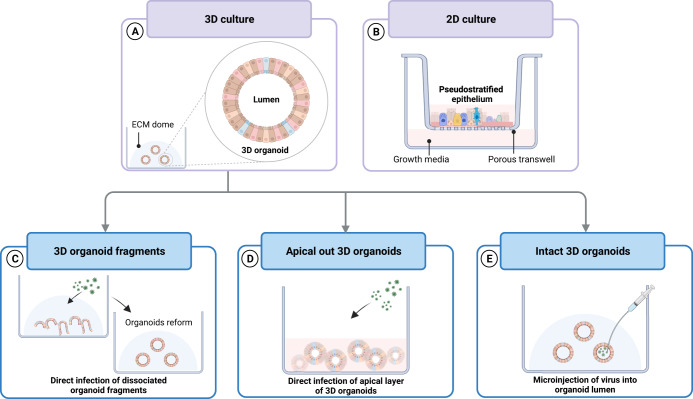
Methods of infecting organoid cultures. Airway organoids are depicted as an example in this figure. Differentiated organoids generated for viral disease studies can be cultured as (**A**). 3D structures embedded in an ECM scaffold or (**B**). a 2D pseudostratified epithelial layer at air-liquid interface. 3D organoids typically require additional processing prior to infection to enable access to the apical layer that is embedded in the interior. This can be achieved through (**C**). enzymatically or mechanically disrupting organoids into fragments and re-embedding into ECM scaffolds prior to infection or (**D**). generating inverted apical-out organoids. Alternatively, viruses can be (**E**). microinjected into the lumen of intact 3D organoids. Figure created with BioRender.

For 3D organoids, the cell surface is often embedded in the interior and inaccessible to viruses. As such, viral infection studies often require additional steps such as disrupting organoids into fragments, precision slicing of organoids^33^, microinjecting viruses into the organoid lumen^34^, or generating inverted organoids that adopt an “apical out” conformation in 3D^35^. In contrast, 2D organoids such as those differentiated on a Transwell at air-liquid interface (ALI), do not have this limitation and thus are frequently used as tools in virology. Comparisons of these available approaches have previously been reviewed^36^ and is illustrated in Fig. 2. In recent years, a wide range of organoid systems have been generated and applied to model human viral diseases.

### Norovirus

Human noroviruses (HuNoVs) are the leading cause of acute and food-borne gastroenteritis worldwide^37^. Unsuccessful attempts in cultivating HuNoVs using primary and continuous cell lines have hindered understanding of disease pathogenesis and development of therapeutics for over three decades. Human intestinal organoids (hIOs) derived from human ASCs provided the first platform that allowed for in vitro HuNoV replication in a reproducible manner, and further aided identification of enterocytes as the predominant intestinal target for infection and replication^38^. Importantly, the study pinpointed bile as a strain-dependent required factor or enhancer for in vitro viral replication, the mechanism of which requires further investigation^38^. hIOs also provided a platform for assessing the effectiveness of virus inactivation strategies as disease countermeasures against different HuNoV strains^39^. Consistent with findings from animal and human studies^40,41^, hIOs demonstrated strain-dependent HuNoV binding specificity for histo-blood group antigens expressed on the mucosal surface of the gastrointestinal tract, matchedness of which was necessary to enable viral replication in vitro^42^. Following successes in HuNoVs studies, hIOs have also been adopted to compare infectivity and host responses elicited by other enteric viruses such as human rotavirus^43^, echovirus (E11)^44^, coxsackie B virus (CVB)^45^ and enterovirus 71^46^. In contrast to observations from infected 2D intestinal cell lines conducted in the same study, infectivity and profile of inflammatory responses mounted by each enteric virus in hIOs were distinct. In particular, CVB and E11 were able to replicate in enteroids with greater efficiency, but robust induction of transcripts for ISGs, cytokines and chemokines associated with antiviral signaling was observed only following infection with E11^47^, collectively demonstrating the promise of hIOs as a human relevant model to unveil critical host-pathogen interactions at the gastro-intestinal cellular barrier.

### Influenza virus

Influenza A viruses (IAV) exhibit a diverse host range and display remarkable capacity to rapidly evolve under selection pressure and adapt to new hosts. IAVs possess a segmented genome and a low fidelity RNA polymerase, which continuously generate reassortants and antigenic variants that have the potential to cross species barriers and cause epidemics, and even pandemics. However, robust in vitro models for predicting zoonotic spillover potential of animal harbored IAVs had previously been lacking. A potential recourse for this is human ASC-derived airway organoids (AOs) that morphologically and functionally resemble human airways at near physiological levels. 2D AOs differentiated under defined conditions demonstrated the ability to distinguish the replicative capacity of human-infective IAVs from poorly human-infective avian and swine subtypes^48^. Importantly, high levels of serine proteases including TMPRSS2, TMPRSS4, HAT and matriptase, which conduct proteolytic cleavage of the virus surface hemagglutinin preceding viral-host membrane fusion, were expressed intrinsically upon AO differentiation^48^. Another study comparing replication capacity and host innate immune responses elicited by avian H5 and H7 viruses isolated from human patients, and human pandemic H1N1, identified higher replication capacity of H1N1 and avian H7N9 in AO cultures, whereas secretion of pro-inflammatory cytokines such as IL-6 and IFN-β were most pronounced following infection with high-pathogenic avian (HPAI) H5N1^49^, for which cytokine dysregulation has been postulated to contribute to severe disease phenotypes in human patients. For H7 subtypes, higher replication capacity was displayed by the human-infective H7N9/Ah strain compared to the poorly human-infective H7N2 in AO^49^. These findings were consistent with observations in ex vivo human bronchus explants. In another study, human AO were used to assess the spillover risk of recent HPAI avian H5N6 and H5N8 isolates. Lower replication competence of H5N6/H5N8 isolates compared to human pandemic H1N1 and H5N1 in AO epithelial cells indicated low likelihood of these strains in contributing to severe disease in humans^50^. Taken together, these studies have substantiated the promise of using AO platforms to assess viral tropism and infectivity in human for pandemic risk assessment of zoonotic IAVs.

Owing to the rapidly evolving nature of IAVs, seasonal influenza vaccine formulations need to be updated annually. Quadrivalent and trivalent inactivated vaccines (IIVs), which are most commonly administered around the world, contain only antigens representative of human IAV and IBV strains, with limited coverage for zoonotic subtypes. While exploration of novel vaccine modalities that can provide broad spectrum protection are underway, majority of pre-clinical vaccine assessments are conducted in mice or non-human primates, and observed responses are often poorly predictive of those in humans^51,52^. Primary human tonsil organoids were recently explored in proof-of-concept studies as a platform for evaluating humoral responses to influenza vaccines. Tonsil organoids cultured in vitro were able to functionally retain germinal centre (GC) features, including production of antigen specific antibodies, somatic hypermutation and affinity maturation upon stimulation with a live-attenuated influenza vaccine (LAIV)^53^. In another study, tonsil organoids isolated and expanded from a single individual established distinct cellular and antibody dynamics following stimulation by different antigen modalities originating from IIVs, LAIVs and IAVs, and has been proposed as a blueprint to profile in vivo immune responses in future clinical trials without the need for experimental animals^54^.

### SARS-CoV-2

The COVID-19 pandemic has heralded an unprecedented volume of research activity over a short period of time. Notably, significant insights into SARS-CoV-2 tropism, replication kinetics and mechanisms of disease pathogenesis were elucidated using organoids. Consistent with findings from human COVID-19 cases^55,56^, ciliated cells expressing ACE2 and TMPRSS2 were identified as the primary cellular target during initial infection in differentiated AOs derived from resected human airway tissues^57^. In severe COVID-19 cases, patients develop acute respiratory distress syndrome that is characterized by diffuse alveolar damage and formation of hyaline membranes that hinders gas exchange^58,59^. A recent study in a primary nasal epithelial organoid model identified that SARS-CoV-2 binds to ACE2 receptors expressed on the surface of motile cilia and hijacks the host cell to trigger formation of elongated apical microvilli, which are used to traverse viral progeny across the mucus layer^60^. Importantly, depletion of epithelial cilia in this model inhibited SARS-CoV-2 infection, suggesting motile cilia as a novel target for blocking entry and spread of viruses in the airway^60^. Due to the lack of clinical material from early infection timepoints, characterization of COVID-19 pathophysiology in the distal lung during early infection has been a challenge. Studies using 3D distal lung organoids have aided identification of club cells as a cellular target in the lower airways^61^. Further, transcriptomic and histological analyses on alveolospheres cultured from primary human lung tissue have shed light on crucial constituents of the inflammatory state upon infection of the alveoli, including upregulation of interferon-mediated responses and apoptosis, as well as a loss of mature AT2 phenotype and AT2 cell death, as indicated by downregulated expression of genes encoding for surfactant proteins^62–65^, which corroborates with clinical findings in COVID-19 lung tissues^66–68^.

Though COVID-19 primarily presents clinically as a respiratory disease, systemic symptoms that manifest during early infection or consequently as disease sequelae have also been reported widely^69^. Moreover, as ACE2 is expressed across various mammalian tissues, SARS-CoV-2 can potentially spread to other organs in the body following initial infection of the respiratory tract. Enterocytes in the intestinal epithelium, which express ACE2 abundantly, were shown to support robust SARS-CoV-2 replication in a small intestine organoid (hSIO) model. Genetic signatures induced by infection in hSIO included cytokines and IFN-stimulated genes associated with IFN type I and III responses^70,71^, which may contribute to gastrointestinal inflammation and in part give rise to gastrointestinal symptoms observed in COVID-19 patients^72^. The type II transmembrane serine protease (TTSP)-dependent entry of SARS-CoV-2 had been identified early during the pandemic in a human AO model, which further pinpointed the multibasic cleavage motif (MBCS) on the virus spike protein to be an adaptation feature that facilitates efficient TTSP usage^73^. Importantly, loss of the MBCS motif is observed upon viral propagation in cells that lack TTSPs, but not in AOs differentiated at ALI^74^. A follow-up study conducted in CRISPR/Cas9 modified hIOs further demonstrated that SARS-CoV-2, as well as MERS-CoV and SARS-CoV, specifically utilize TMPRSS2 for entry, making it an attractive target for pan-coronavirus therapeutics. During the emergence of the Omicron variant, several studies reported a switch in viral entry pathway towards TMPRSS2-independent endosomal entry^75,76^. This was later demonstrated in AOs and CRISPR/Cas9 modified hSIOs to be a cell line-specific phenomenon, as TTSP-mediated entry was still efficiently utilized by Omicron in organoids^77^.

Besides elucidating host-virus interaction and pathophysiological mechanisms, organoids have also shown promise as a translational platform for drug efficacy determination and identification of novel therapeutics for SARS-CoV-2. In particular, cardiac, lung and hIOs have been adopted for drug screening, from which a broad spectrum of SARS-CoV-2 entry inhibitors^78^, replication suppressors^63,79^, as well as cardioprotective drugs^80^ that could be utilized as COVID-19 therapeutics had been identified. Additionally, innovative efforts to combine organoid models with gene editing techniques such as CRISPR/Cas9 to identify host genetic factors implied in SARS-CoV-2 infection have recently been established in the form of a CRISPR-knockout biobank^81^. Combination of CRISPR/Cas9 technology with human PSC-derived hepatocyte and blood vessel organoids have also been implemented in studies for viruses beyond SARS-CoV-2, such as filoviruses, which have identified the guanine nucleoside exchange factor CCZ1 as an essential host factor that controls early replication of Ebola and Marburg viruses^82^. Such technique could be applied to future studies of other novel and emerging viruses to enable rapid characterization of host factors involved in pathogen interaction, and identify targets for antiviral development. Additionally, organoids can also be readily cultured from diverse host species, thus hold promise to model differences in virus susceptibility between hosts. New animal derived organoid models ranging from snake venom glands^83^ to bat intestines^70^ are being established rapidly, some of which had already been applied to study virus tropism and host responses, such as for SARS-CoV-2^70^.

### Zika virus

Since its identification in Africa, the mosquito-borne Zika virus (ZIKV) has spread extensively across the globe. Global footprint of ZIKV has increased drastically in the last two decades, and has caused large outbreaks in the Yap Islands in 2007^84^, in French Polynesia in 2013^85^ and in South America in 2015^86^. While majority of ZIKV cases are asymptomatic or mildly symptomatic, concern of ZIKV-associated severe pathogenic effects became prominent during the 2015 outbreak in Brazil, where a substantial number of infections during pregnancy was associated with neurological abnormalities and in particular, microencephaly in newborns^87^. Due to the lack of accessibility to live infected human fetal tissues, clinical understanding of ZIKV pathogenesis in the fetal central nervous system were limited. Using human PSC-derived cerebral organoids and pregnant mouse models, multiple groups have demonstrated that ZIKV infection results in disruption of the cerebral cortical layers and decline in proliferative zones, as well as reduced levels of functional neurones^88,89^. In accordance with these findings, another study that used brain region-specific organoids grown in spinning bioreactors showed that both Asian and African ZIKV strains preferentially and productively infected neural progenitors and triggered premature differentiation^90^, which closely resembled infection patterns observed in human fetuses^91^. Subsequent studies in human PSC-derived brain organoids identified alterations in the DNA methylome of neural progenitors and differentiated neurons following ZIKV infection at genes implicated in neuropsychiatric disorders^92^, suggesting neuropsychiatric complications as a potential downstream effect of fetal ZIKV infection. Additionally, human cortical organoids have been adopted in drug repurposing screens for therapeutics that can limit ZIKV induced progenitor cell death from FDA-approved drug libraries^93^ as well as in characterizing the mechanism of action of novel anti-ZIKV compounds^94^.

### Limitations of organoid platforms in virology

As organoid platforms gain increasing popularity as in vitro tools for modeling viral diseases, it is important to recognize the advantages and address the limitations of each organoid system to achieve improved models with optimal physiological relevance, and increase translational applicability of findings. For example, ASC-derived organoids can well represent the architecture and functional aspects of the adult tissue epithelium, and stably retain epigenetic signatures from the original tissue throughout passages^95^. As such, they are able to model responses to viral infections within different genetic backgrounds and disease states. However, ASC-organoids generally lack representation beyond epithelial phenotypes, which include stromal, immune and vascular niches, and thus are of limited representativeness of epithelial-microenvironment interactions in the context of viral diseases. On the other hand, PSCs are valuable for culturing organoids from tissues with stem cells that are difficult to access or are of embryonic derivative, such as the brain. PSC-derived organoids typically contain both epithelial and mesenchymal layers, making them of greater complexity than ASC-derived organoids and can support investigations of epithelial-mesenchymal interactions during viral infections. However, even in a fully differentiated state, PSC models often retain cellular phenotypes that are fetal or neonatal, which limits their representativeness of viral-host interactions in adult functional tissues^96,97^.

A shortcoming that is common in both ASC and PSC models is the general lack of immune cell populations, which is pivotal in the context of viral disease modeling, since excessive inflammation or dysfunctional immune responses often play an equally or more important role in inducing host damage than direct infection^58,98,99^. This infection-induced hyperinflammatory state has yet to be thoroughly modeled in any organoid platforms. Another aspect with room for improvement in both models is better definition of growth conditions to achieve greater reproducibility of findings. This may entail adopting culture methods that can limit artefacts from ECM derivatives, such as inflammatory proteins, which can lead to heterogeneity in organoid formation from study to study^100^. In terms of integrating organoid platforms into translational research, a major challenge is to generate high-throughput platforms that are complementary with organoid technology. In one study, human PSC-derived lung organoid cultured in a 384-well format was used for antiviral screening, and identified FDA approved drugs such as imatinib and mycophenolic acid to be able to inhibit SARS-CoV-2 entry^101^. Other models have additionally incorporated custom-engineered platforms, such as a 96-well microchannel based ALI system, that is designed to be compatible with high resolution in situ imaging and real-time sensing. The platform supported replication of IAV and the human seasonal coronavirus NL63, for which detected viral copies could be reduced using oseltamivir and camostat mesylate respectively^102^. As of present, organoid platforms for certain high-risk pathogens such as causative agents for viral hemorrhagic fevers remain scarce, in part due to the stringency of BSL-4 containment facilities. However, research on these viruses —particularly those featured in the WHO priority list^103^—is critical for epidemic and pandemic preparedness. Finally, since viral disease pathophysiology rarely localizes in a single organ, employing novel strategies to capture inter-organ interactions would be essential to understand systemic effects of viral infections. Approaches to address these existing limitations are currently being explored progressively in organoid research.

### “Next-generation” organoid platforms

#### Immune co-culture

For viral disease studies, devising methods to include immune cell populations in organoid platforms is a critical consideration going forward, since exacerbated or dysfunctional immune responses are thought to underlie severe disease for many viruses, including SARS-CoV-2 ^58,99,104^, HPAI H5N1^105^ and Ebola^106^. Acute cardiac injury was shown to be associated with a significantly greater mortality rate in COVID-19 patients, but causes of myocardial histopathology is poorly understood. Previously, studies from post-mortem heart samples from COVID-19 patients have identified increased inflammatory infiltration of CD11b^+^ and CD68^+^ macrophages^107–110^. To model the effects of macrophage-mediated hyperinflammation in cardiomyocytes (CM), an immuno-cardiac co-culture platform was generated using human PSC-derived CMs and monocytes/macrophages. The study identified CCL2 as the primary chemokine secreted by CMs to recruit monocytes upon SARS-CoV-2 infection, and presence of macrophages to significantly reduce the incidence of infected CMs^111^. In a separate co-culture study, IL-6 and TNF-ɑ generated by recruited macrophages during SARS-CoV-2 infection resulted in increased levels reactive oxygen species and apoptosis of CMs. Inhibition of IL-6 and TNF-ɑ through drug blockage of the JAK/STAT pathway was able to protect CMs from macrophage-induced hyperinflammation^112^.

A recent complex lung organoid model generated from intact human lung fragments was shown to retain differentiated lung epithelial lineages, mesenchymal components as well as tissue-resident immune cell subsets. Innate and adaptive immune responses following SARS-CoV-2 infection of the complex organoid mirrored clinical observations in vivo^113^. Importantly, virus-specific memory T cell responses was observed following infection in lung samples from SARS-CoV-2 seropositive donors, demonstrating the capacity for mounting of lung resident T cell memory in absence of peripheral lymphoid components in this co-culture system. While maintaining immune cell longevity and phenotype preservation is a major challenge for organoid immune co-cultures, the study observed that T cell-tropic cytokines helped to sustain organoid immune content over time^114^.

Studies of other respiratory viruses beyond SARS-CoV-2 have also adopted immune co-culture models. Differentiated nasal epithelium co-cultured with human PBMCs were used to characterize epithelium-leukocyte crosstalk during exposure to the influenza H3N2 strain, identifying monocytes, NK cells and innate T cells as the first to be activated at early stages of infection^115^. By partitioning epithelial cells and PBMCs in culture with a Transwell insert, direct PBMC infection was prevented, allowing for identification of immune responses triggered by soluble factors that are released by the infected epithelium^115^. Co-cultures of human pulmonary organoids and neutrophils have also been established previously to investigate neutrophil migration patterns in the context of inflammation, and been adapted to study neutrophil-epithelium interactions during RSV infection, identifying cytokines such as IP-10 and RANTES to have a crucial role in recruiting leukocytes during early infection^34^.

**Fig. 3:**
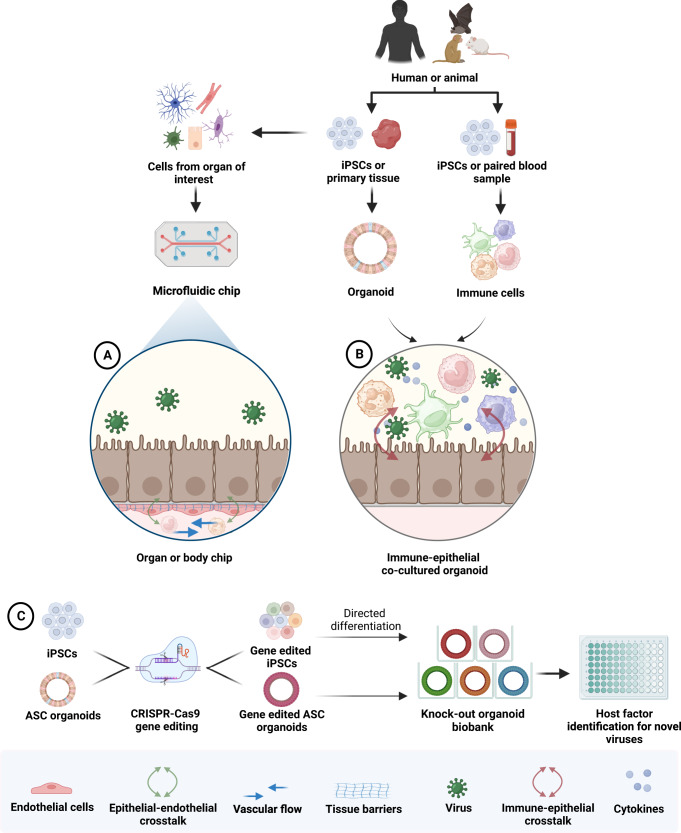
“Next generation” organoid models for virus research. **A** Organ-on-a-chip. Microfluidic-based single or multiple organ-on-a-chip platforms engineered to contain vascular flow and spatiotemporal mechanical and chemical gradients can be used to model complex interactions between the epithelium, stroma, and circulating immune components during virus infection. Multi-organ “body” chips can additionally capture inter-organ crosstalk during infection to model systemic disease. **B** Immune co-culture. ASC or PSC organoids can be cultured with immune cells isolated from a paired-blood sample or generated from PSCs to capture immune-epithelial crosstalk during viral infections. **C** CRISPR/Cas9-gene edited organoids. Knock-out organoid biobanks can be used to screen for and identify host factors utilized by novel viruses for entry. Biobanks can be generated from CRISPR/Cas9-edited ASC organoids, or edited iPSC progenitors that are subsequently expanded clonally into PSC organoids. Figure created with BioRender.

Recently, a co-culture study established using PSC-derived liver organoids (LOs) comprised of hepatocytes and Kupffer-like cells, and macrophages from the same donor, was used to elucidate the role of hepatitis C virus (HCV) infection in the pathogenesis of non-alcoholic fatty liver disease (NAFLD)^116^. In particular, infection with HCV upregulated host lipogenesis resulting in fatty acid accumulation in LOs, which was accentuated in the presence of macrophages. Moreover, therapeutics that are used to target non-alcoholic steatohepatitis and are currently in late-stage clinical trials elicited similar outcomes in the LO platform and in human cohorts, demonstrating the potential for the co-culture system to be used for future evaluation of NAFLD pathogenesis, and potentially other chronic liver diseases, as well as for evaluation of therapeutic efficacy^116^. Currently, novel organoids platforms to model immune responses to viral infections in the lymphoid microenvironment are also being established. Lymphoid organoids proliferated from murine splenocytes has established a basis for in vitro induction of GC-like B cells, which could be applied to elucidate mechanisms of mounting humoral B cell immunity during viral infections in humans, as well as development of vaccines and antibody-based immunotherapy against viral diseases^117^. As immune co-culture organoid systems advance rapidly and become increasingly incorporated into viral disease studies, additional considerations need to be accounted for, such as high variability of immune responses between individuals to viral infections, depending on their immunological history^118^. For this, individualized co-culture platforms generated from patient tissues and paired-blood samples can be utilized (Fig. 3). Additionally, future studies on immunological responses to viral infection will have to optimize co-culture systems to faithfully recapitulate immune cell states during infection, and support long-term preservation of immune cells in culture.

#### Organ-on-a-chip

While existing organoid platforms show promise in representing viral disease pathogenesis in individual tissues or organs, they lack the ability to recapitulate systemic disease, which involves signaling cues and crosstalk between epithelium components and their surrounding microenvironment. A possible avenue to bridge these limitations is microfluidics-based organ chips. Minimalistic organ chips are generally composed of cells from a tissue of interest, an ECM interface and a neighboring vascular and/or connective tissues which are seeded onto interconnected flow-through chambers of a microfluidic device^119–121^. Since the first development of a lung alveolus organ chip, numerous designs have become available at pre-commercial and commercial stages, allowing for prototyping of cellular, tissue, and organ level responses for different viral diseases^122–125^.

Perfused human gut chip platforms have been applied to study enteric virus infection. In addition to an intestinal epithelial layer, a model additionally incorporated a vascular channel that is separated from the epithelium by a porous matrix-coated membrane to generate continuous fluid flow through the intestinal lumen^126^. Under consistent perfusion and cyclic mechanical strain, spontaneous formation of a differentiated 3D epithelial layer with villus-like structures containing proliferative crypts, mucus-secreting, Paneth and enteroendocrine cells that resemble the in vivo intestinal epithelium was enabled. Reconstituted human airways consisting of differentiated airway epithelium cultured in parallel channels with pulmonary microvascular components was used to recapitulate epithelial and endothelium dysfunction as in human airways in vivo when inoculated with IAV. The airway chip has also been utilized to study human rhinovirus-induced exacerbation of asthma, characterizing immune cell transendothelial migration and hallmark inflammatory markers of the disease state^127^. Perfusable, endothelialized microvessel chips have shown promise for studying vascular dysfunction resulting from infection with haemorrhagic viruses. In addition to identifying vascular integrity loss and the resultant increase in vascular permeability following infusion of Ebola virus-like particles, the platform also identified activation of the Rho/ROCK pathway to be a mediator of cytoskeleton remodeling which resulted in albumin leakage from the engineered vessels^128^. These chip platforms can potentially offer a new avenue for uncovering pathophysiological mechanisms and provide a basis for identifying candidate therapeutics for haemorrhagic viruses, which otherwise would typically require non-human primate model studies in specialized animal BSL-4 facilities.

Organ-on-a-chip technology can imbue drawbacks of existing organoid platforms, such as presence tissue barriers, vascular flow, spatiotemporal mechanical and chemical gradients as well as recruitment of circulating immune cells (Fig. 3). Recent efforts have further built upon existing chip technology to integrate multiple organs on the same platform to form “body chips”, allowing simulation of inter-organ communication and systemic function^129,130^. However, existing microfluidic chip platforms still have drawbacks, particularly since they often contain immortalized cell lines. Increasing effort towards advancing chip platforms to contain organoid-derived cells would help attain greater physiological likedness. Achieving in vitro platforms that can model viral disease to high physiological relevance with reproducibility is of great priority, particularly as organoids and organ-on-a-chip models are gaining increased traction in translational research as a substitute for animal testing^131,132^. Integrating human-derived stem cells into chip compositions could also allow for generation of individualized organ chips composed of homogenous genotypes, which can serve as platforms for personalized therapeutic development.

## Conclusion

Over the past decade, organoid platforms have contributed important findings to virus research by addressing critical knowledge gaps in viral-host interaction and disease pathophysiology. These models have shown to be advantageous in their tractability and high human physiological relevance compared to other traditional experimental platforms. As organoids become increasingly prominent as in vitro models for virology studies, crucial shortcomings that exist in platforms need to be addressed. Novel approaches to improve existing organoid systems are currently being explored widely. Next-generation organoid platforms such as immune co-culture models that can emulate epithelial-immune crosstalk during viral infections, microfluidics-based organ chips that can capture systemic effects of viral disease, and CRISPR-edited organoids that allow for precise identification of host targets that modulate viral infections, show great promise to help refine organoid technology to better accommodate viral research studies.

## References

[1] Rulli, M. C. et al. Land-use change and the livestock revolution increase the risk of zoonotic coronavirus transmission from rhinolophid bats. *Nat. Food* **2**, 409–416 (2021). 37118224 10.1038/s43016-021-00285-x

[2] Carlson, C. J. et al. Climate change increases cross-species viral transmission risk. *Nature* **607**, 555–562 (2022). 35483403 10.1038/s41586-022-04788-w

[3] Jones, K. et al. Global trends in emerging infectious diseases. *Nature* **451**, 990–993 (2008). 18288193 10.1038/nature06536 PMC5960580

[4] Scherer, W. F., Syverton, J. T. & Gey, G. O. Studies on the propagation in vitro of poliomyelitis viruses. *J. Exp. Med.* **97**, 695–710 (1953). 13052828 10.1084/jem.97.5.695 PMC2136303

[5] Sasaki, M. et al. SARS-CoV-2 variants with mutations at the S1/S2 cleavage site are generated in vitro during propagation in TMPRSS2-deficient cells. *PLOS Pathog.* **17**, e1009233 (2021). 33476327 10.1371/journal.ppat.1009233 PMC7853460

[6] Le Sage, V. et al. Cell-culture adaptation of H3N2 influenza virus impacts acid stability and reduces airborne transmission in ferret model. *Viruses* **13**, 719 (2021). 33919124 10.3390/v13050719 PMC8143181

[7] Johnson, B. A. et al. Loss of furin cleavage site attenuates SARS-CoV-2 pathogenesis. *Nature* **591**, 293–299 (2021). 33494095 10.1038/s41586-021-03237-4 PMC8175039

[8] Bankamp, B., Fontana, J. M., Bellini, W. J. & Rota, P. A. Adaptation to cell culture induces functional differences in measles virus proteins. *Virol. J.* **5**, 129 (2008). 18954437 10.1186/1743-422X-5-129 PMC2582235

[9] Hayflick, L. & Moorhead, P. S. The serial cultivation of human diploid cell strains. *Exp. Cell Res.* **25**, 585–621 (1961). 13905658 10.1016/0014-4827(61)90192-6

[10] van den Hoogen, B. G. et al. A newly discovered human pneumovirus isolated from young children with respiratory tract disease. *Nat. Med.* **7**, 719–724 (2001). 11385510 10.1038/89098 PMC7095854

[11] Fouchier, R. A. M. et al. Koch’s postulates fulfilled for SARS virus. *Nature* **423**, 240–240 (2003). 12748632 10.1038/423240a PMC7095368

[12] Mukherjee, P. et al. Role of animal models in biomedical research: a review. *Lab. Anim Res.* **38**, 18 (2022). 35778730 10.1186/s42826-022-00128-1 PMC9247923

[13] Chaudhari, U. et al. An overview of preclinical animal models for SARS-CoV-2 pathogenicity. *Indian J. Med. Res.* **153**, 17 (2021). 33818465 10.4103/ijmr.IJMR_3215_20 PMC8184076

[14] Carrion, R. et al. Lassa virus infection in experimentally infected marmosets: liver pathology and immunophenotypic alterations in target tissues. *J. Virol.* **81**, 6482–6490 (2007). 17409137 10.1128/JVI.02876-06 PMC1900113

[15] Margine, I. & Krammer, F. Animal models for influenza viruses: implications for universal vaccine development. *Pathogens* **3**, 845–874 (2014). 25436508 10.3390/pathogens3040845 PMC4282889

[16] Rothenburg, S. & Brennan, G. Species-specific host–virus interactions: implications for viral host range and virulence. *Trends Microbiol.* **28**, 46–56 (2020). 31597598 10.1016/j.tim.2019.08.007 PMC6925338

[17] Gu, H. et al. Adaptation of SARS-CoV-2 in BALB/c mice for testing vaccine efficacy. *Science* **369**, 1603–1607 (2020). 32732280 10.1126/science.abc4730 PMC7574913

[18] Roberts, A. et al. A mouse-adapted SARS-coronavirus causes disease and mortality in BALB/c mice. *PLoS Pathog.* **3**, e5 (2007). 17222058 10.1371/journal.ppat.0030005 PMC1769406

[19] Chan, M. et al. Generation and characterization of a mouse-adapted makona variant of Ebola virus. *Viruses* **11**, 987 (2019). 31717793 10.3390/v11110987 PMC6893688

[20] Menachery, V. D. et al. SARS-like WIV1-CoV poised for human emergence. *Proc. Natil. Acad. Sci.* **113**, 3048–3053 (2016). 10.1073/pnas.1517719113 PMC4801244 26976607

[21] Sun, S.-H. et al. A mouse model of SARS-CoV-2 infection and pathogenesis. *Cell Host Microbe* **28**, 124–133.e4 (2020). 32485164 10.1016/j.chom.2020.05.020 PMC7250783

[22] Chu, H., Chan, J. F. & Yuen, K. Y. Animal models in SARS-CoV-2 research. *Nat. Methods* **19**, 392–394 (2022). 35396468 10.1038/s41592-022-01447-w

[23] Lancaster, M. A. & Knoblich, J. A. Organogenesis in a dish: modeling development and disease using organoid technologies. *Science* **345**, 1247125 (2014). 25035496 10.1126/science.1247125

[24] Sato, T. & Clevers, H. Growing self-organizing mini-guts from a single intestinal stem cell: mechanism and applications. *Science* **340**, 1190–1194 (2013). 23744940 10.1126/science.1234852

[25] Chiu, M. C. et al. A bipotential organoid model of respiratory epithelium recapitulates high infectivity of SARS-CoV-2 Omicron variant. *Cell Discov.* **8**, 57 (2022). 35710786 10.1038/s41421-022-00422-1 PMC9203776

[26] Braverman, J. & Yilmaz, Ö. H. From 3D organoids back to 2D enteroids. *Dev. Cell* **44**, 533–534 (2018). 29533766 10.1016/j.devcel.2018.02.016

[27] Thorne, C. A. et al. Enteroid monolayers reveal an autonomous WNT and BMP circuit controlling intestinal epithelial growth and organization. *Dev. Cell* **44**, 624–633.e4 (2018). 29503158 10.1016/j.devcel.2018.01.024 PMC5849535

[28] Wang, Y. et al. Self-renewing monolayer of primary colonic or rectal epithelial cells. *Cell. Mol. Gastroenterol. Hepatol.* **4**, 165–182.e7 (2017). 29204504 10.1016/j.jcmgh.2017.02.011 PMC5710741

[29] Clevers, H. Modeling development and disease with organoids. *Cell* **165**, 1586–1597 (2016). 27315476 10.1016/j.cell.2016.05.082

[30] Takahashi, K. & Yamanaka, S. Induction of pluripotent stem cells from mouse embryonic and adult fibroblast cultures by defined factors. *Cell* **126**, 663–676 (2006). 16904174 10.1016/j.cell.2006.07.024

[31] Barker, N. et al. Identification of stem cells in small intestine and colon by marker gene Lgr5. *Nature* **449**, 1003–1007 (2007). 17934449 10.1038/nature06196

[32] Sato, T. et al. Single Lgr5 stem cells build crypt-villus structures in vitro without a mesenchymal niche. *Nature* **459**, 262–265 (2009). 19329995 10.1038/nature07935

[33] Watanabe, M. et al. Self-organized cerebral organoids with human-specific features predict effective drugs to combat zika virus infection. *Cell Rep.* **21**, 517–532 (2017). 29020636 10.1016/j.celrep.2017.09.047 PMC5637483

[34] Sachs, N. et al. Long‐term expanding human airway organoids for disease modeling. *EMBO J.* **38**, e100300 (2019). 30643021 10.15252/embj.2018100300 PMC6376275

[35] Stroulios, G. et al. Apical-out airway organoids as a platform for studying viral infections and screening for antiviral drugs. *Sci. Rep.* **12**, 7673 (2022). 35538146 10.1038/s41598-022-11700-z PMC9089294

[36] Aguilar, C. et al. Organoids as host models for infection biology – a review of methods. *Exp. Mol. Med.* **53**, 1471–1482 (2021). 34663936 10.1038/s12276-021-00629-4 PMC8521091

[37] Lopman, B. A., Steele, D., Kirkwood, C. D. & Parashar, U. D. The vast and varied global burden of norovirus: prospects for prevention and control. *PLOS Med.* **13**, e1001999 (2016). 27115709 10.1371/journal.pmed.1001999 PMC4846155

[38] Ettayebi, K. et al. Replication of human noroviruses in stem cell-derived human enteroids. *Science* **353**, 1387–1393 (2016). 27562956 10.1126/science.aaf5211 PMC5305121

[39] Costantini, V. et al. Human norovirus replication in human intestinal enteroids as model to evaluate virus inactivation. *Emerg. Infect. Dis.* **24**, 1453–1464 (2018). 30014841 10.3201/eid2408.180126 PMC6056096

[40] Rockx, B. H. G., Bogers, W. M. J. M., Heeney, J. L., van Amerongen, G. & Koopmans, M. P. G. Experimental norovirus infections in non‐human primates. *J. Med. Virol.* **75**, 313–320 (2004). 10.1002/jmv.20273 15602728

[41] Cheetham, S. et al. Pathogenesis of a genogroup II human norovirus in Gnotobiotic Pigs. *J. Virol.* **80**, 10372–10381 (2006). 17041218 10.1128/JVI.00809-06 PMC1641747

[42] Zhang, D. et al. Human intestinal organoids express histo-blood group antigens, bind norovirus VLPs, and support limited norovirus replication. *Sci. Rep.* **7**, 12621 (2017). 28974702 10.1038/s41598-017-12736-2 PMC5626734

[43] Finkbeiner, S. R. et al. Stem cell-derived human intestinal organoids as an infection model for rotaviruses. *mBio* **3**, e00159–12 (2012). 22761392 10.1128/mBio.00159-12 PMC3398537

[44] Saxena, K. et al. Human intestinal enteroids: a new model to study human rotavirus infection, host restriction, and pathophysiology. *J. Virol.* **90**, 43–56 (2016). 26446608 10.1128/JVI.01930-15 PMC4702582

[45] Villenave, R. et al. Human gut-on-a-chip supports polarized infection of Coxsackie B1 virus in vitro. *PLOS ONE* **12**, e0169412 (2017). 28146569 10.1371/journal.pone.0169412 PMC5287454

[46] Tsang, J. O.-L. et al. Development of three-dimensional human intestinal organoids as a physiologically relevant model for characterizing the viral replication kinetics and antiviral susceptibility of enteroviruses. *Biomedicines* **9**, 88 (2021). 33477611 10.3390/biomedicines9010088 PMC7831294

[47] Drummond, C. G. et al. Enteroviruses infect human enteroids and induce antiviral signaling in a cell lineage-specific manner. *Proc. Natl. Acad. Sci.* **114**, 1672–1677 (2017). 28137842 10.1073/pnas.1617363114 PMC5320971

[48] Zhou, J. et al. Differentiated human airway organoids to assess infectivity of emerging influenza virus. *Proc. Natl. Acad. Sci.* **115**, 6822–6827 (2018). 29891677 10.1073/pnas.1806308115 PMC6042130

[49] Hui, K. P. Y. et al. Tropism, replication competence, and innate immune responses of influenza virus: an analysis of human airway organoids and ex-vivo bronchus cultures.*Lancet Respir. Med.* **6**, 846–854 (2018). 30001996 10.1016/S2213-2600(18)30236-4

[50] Bui, C. H. T. et al. Risk assessment for highly pathogenic Avian Influenza A(H5N6/H5N8) Clade 2.3.4.4 viruses. *Emerg. Infect. Dis.* **27**, 2619–2627 (2021). 34545790 10.3201/eid2710.210297 PMC8462306

[51] Rivera, J. & Tessarollo, L. Genetic background and the dilemma of translating mouse studies to humans. *Immunity* **28**, 1–4 (2008). 18199409 10.1016/j.immuni.2007.12.008

[52] Watkins, D. I., Burton, D. R., Kallas, E. G., Moore, J. P. & Koff, W. C. Nonhuman primate models and the failure of the Merck HIV-1 vaccine in humans. *Nat. Med.* **14**, 617–621 (2008). 18535579 10.1038/nm.f.1759 PMC3697853

[53] Wagar, L. E. et al. Modeling human adaptive immune responses with tonsil organoids. *Nat. Med.* **27**, 125–135 (2021). 33432170 10.1038/s41591-020-01145-0 PMC7891554

[54] Kastenschmidt, J. M. et al. Influenza vaccine format mediates distinct cellular and antibody responses in human immune organoids. *Immunity* **56**, 1910–1926.e7 (2023). 37478854 10.1016/j.immuni.2023.06.019 PMC10433940

[55] Hou, Y. J. et al. SARS-CoV-2 reverse genetics reveals a variable infection gradient in the respiratory tract. *Cell* **182**, 429–446.e414 (2020). 32526206 10.1016/j.cell.2020.05.042 PMC7250779

[56] Ahn, J. H. et al. Nasal ciliated cells are primary targets for SARS-CoV-2 replication in the early stage of COVID-19. *J. Clin. Investig.* **131**, e148517 (2021). 34003804 10.1172/JCI148517 PMC8245175

[57] Milewska, A. et al. Replication of severe acute respiratory syndrome Coronavirus 2 in human respiratory epithelium. *J. Virol.* **94**, e00957–20 (2020). 32434888 10.1128/JVI.00957-20 PMC7375387

[58] Lamers, M. M. & Haagmans, B. L. SARS-CoV-2 pathogenesis. *Nat. Rev. Microbiol.* **20**, 270–284 (2022). 35354968 10.1038/s41579-022-00713-0

[59] Konopka, K. E. et al. Diffuse alveolar damage (DAD) resulting from coronavirus disease 2019 infection is morphologically indistinguishable from other causes of DAD. *Histopathology* **77**, 570–578 (2020). 32542743 10.1111/his.14180 PMC7323403

[60] Wu, C.-T. et al. SARS-CoV-2 replication in airway epithelia requires motile cilia and microvillar reprogramming. *Cell* **186**, 112–130.e20 (2023). 36580912 10.1016/j.cell.2022.11.030 PMC9715480

[61] Salahudeen, A. A. et al. Progenitor identification and SARS-CoV-2 infection in human distal lung organoids. *Nature* **588**, 670–675 (2020). 33238290 10.1038/s41586-020-3014-1 PMC8003326

[62] Katsura, H. et al. Human lung stem cell-based Alveolospheres provide insights into SARS-CoV-2-mediated interferon responses and pneumocyte dysfunction. *Cell Stem Cell* **27**, 890–904.e8 (2020). 33128895 10.1016/j.stem.2020.10.005 PMC7577733

[63] Mulay, A. et al. SARS-CoV-2 infection of primary human lung epithelium for COVID-19 modeling and drug discovery. *Cell Rep.* **35**, 109055 (2021). 33905739 10.1016/j.celrep.2021.109055 PMC8043574

[64] Youk, J. et al. Three-dimensional human alveolar stem cell culture models reveal infection response to SARS-CoV-2. *Cell Stem Cell* **27**, 905–919.e10 (2020). 33142113 10.1016/j.stem.2020.10.004 PMC7577700

[65] Huang, J. et al. SARS-CoV-2 infection of pluripotent stem cell-derived human lung alveolar Type 2 cells elicits a rapid epithelial-intrinsic inflammatory response. *Cell Stem Cell* **27**, 962–973.e7 (2020). 32979316 10.1016/j.stem.2020.09.013 PMC7500949

[66] Chen, J., Wu, H., Yu, Y. & Tang, N. Pulmonary alveolar regeneration in adult COVID-19 patients. *Cell Res.* **30**, 708–710 (2020). 32632255 10.1038/s41422-020-0369-7 PMC7338112

[67] Delorey, T. M. et al. COVID-19 tissue atlases reveal SARS-CoV-2 pathology and cellular targets. *Nature* **595**, 107–113 (2021). 33915569 10.1038/s41586-021-03570-8 PMC8919505

[68] Melms, J. C. et al. A molecular single-cell lung atlas of lethal COVID-19. *Nature* **595**, 114–119 (2021). 33915568 10.1038/s41586-021-03569-1 PMC8814825

[69] Ballering, A. V., van Zon, S. K. R., Olde Hartman, T. C. & Rosmalen, J. G. M. Persistence of somatic symptoms after COVID-19 in the Netherlands: an observational cohort study. *Lancet* **400**, 452–461 (2022). 35934007 10.1016/S0140-6736(22)01214-4 PMC9352274

[70] Zhou, J. et al. Infection of bat and human intestinal organoids by SARS-CoV-2. *Nat. Med.* **26**, 1077–1083 (2020). 32405028 10.1038/s41591-020-0912-6

[71] Lamers, M. M. et al. SARS-CoV-2 productively infects human gut enterocytes. *Science* **369**, 50–54 (2020). 32358202 10.1126/science.abc1669 PMC7199907

[72] Gu, J., Han, B. & Wang, J. COVID-19: gastrointestinal manifestations and potential Fecal–Oral Transmission. *Gastroenterology* **158**, 1518–1519 (2020). 32142785 10.1053/j.gastro.2020.02.054 PMC7130192

[73] Mykytyn, A. Z. et al. SARS-CoV-2 entry into human airway organoids is serine protease-mediated and facilitated by the multibasic cleavage site. *eLife* **10**, e64508 (2021). 33393462 10.7554/eLife.64508 PMC7806259

[74] Lamers, M. M. et al. Human airway cells prevent SARS-CoV-2 multibasic cleavage site cell culture adaptation. *eLife* **10**, e66815 (2021). 33835028 10.7554/eLife.66815 PMC8131099

[75] Meng, B. et al. Altered TMPRSS2 usage by SARS-CoV-2 omicron impacts infectivity and fusogenicity. *Nature* **603**, 706–714 (2022). 35104837 10.1038/s41586-022-04474-x PMC8942856

[76] Willett, B. J. et al. SARS-CoV-2 Omicron is an immune escape variant with an altered cell entry pathway.*Nat Microbiol.* **7**, 1161–1179 (2022). 35798890 10.1038/s41564-022-01143-7 PMC9352574

[77] Mykytyn, A. Z. et al. SARS-CoV-2 Omicron entry is type II transmembrane serine protease-mediated in human airway and intestinal organoid models. *J. Virol.* **97**, e0085123 (2023). 37555660 10.1128/jvi.00851-23 PMC10506477

[78] Han, Y. et al. Identification of SARS-CoV-2 inhibitors using lung and colonic organoids. *Nature* **589**, 270–275 (2020). 33116299 10.1038/s41586-020-2901-9 PMC8034380

[79] Lee, J.-E. et al. Development of a screening platform to discover natural products active against SARS-CoV-2 infection using lung organoid models. *Biomater. Res.* **27**, 18 (2023). 36855173 10.1186/s40824-023-00357-y PMC9974403

[80] Mills, R. J. et al. BET inhibition blocks inflammation-induced cardiac dysfunction and SARS-CoV-2 infection. *Cell* **184**, 2167–2182.e22 (2021). 33811809 10.1016/j.cell.2021.03.026 PMC7962543

[81] Beumer, J. et al. A CRISPR/Cas9 genetically engineered organoid biobank reveals essential host factors for coronaviruses. *Nat. Commun.* **12**, 5498 (2021). 34535662 10.1038/s41467-021-25729-7 PMC8448725

[82] Monteil, V. et al. Identification of CCZ1 as an essential lysosomal trafficking regulator in Marburg and Ebola virus infections. *Nat. Commun.* **14**, 6785 (2023). 37880247 10.1038/s41467-023-42526-6 PMC10600203

[83] Post, Y. et al. Snake venom gland organoids. *Cell* **180**, 233–247.e21 (2020). 31978343 10.1016/j.cell.2019.11.038

[84] Lanciotti, R. S. et al. Genetic and serologic properties of Zika virus associated with an epidemic, Yap State, Micronesia, 2007. *Emerg. Infect. Dis.* **14**, 1232–1239 (2008). 18680646 10.3201/eid1408.080287 PMC2600394

[85] Oehler, E. et al. Zika virus infection complicated by Guillain-Barré syndrome – case report, French Polynesia, December 2013. *Eurosurveillance* **19**, 20720 (2014). 24626205 10.2807/1560-7917.es2014.19.9.20720

[86] Dick, G. W. A., Kitchen, S. F. & Haddow, A. J. Zika Virus (I). Isolations and serological specificity. *Trans. R. Soc. Trop. Med. Hyg.* **46**, 509–520 (1952). 12995440 10.1016/0035-9203(52)90042-4

[87] Kleber de Oliveira, W. et al. Increase in reported prevalence of microcephaly in infants born to women living in areas with confirmed Zika virus transmission during the first trimester of pregnancy — Brazil, 2015. *Morb. Mortal. Weekly Rep.* **65**, 242–247 (2016). 10.15585/mmwr.mm6509e2 26963593

[88] Garcez, P. P. et al. Zika virus impairs growth in human neurospheres and brain organoids. *Science* **352**, 816–818 (2016). 27064148 10.1126/science.aaf6116

[89] Cugola, F. R. et al. The Brazilian Zika virus strain causes birth defects in experimental models. *Nature* **534**, 267–271 (2016). 27279226 10.1038/nature18296 PMC4902174

[90] Qian, X. et al. Brain-region-specific organoids using mini-bioreactors for modeling ZIKV exposure. *Cell* **165**, 1238–1254 (2016). 27118425 10.1016/j.cell.2016.04.032 PMC4900885

[91] Gabriel, E. et al. Recent Zika virus isolates induce premature differentiation of neural progenitors in human brain organoids. *Cell Stem Cell* **20**, 397–406.e5 (2017). 28132835 10.1016/j.stem.2016.12.005

[92] Janssens, S. et al. Zika Virus alters DNA methylation of neural genes in an organoid model of the developing human brain. *mSystems* **3**, e00219–17 (2018). 29435496 10.1128/mSystems.00219-17 PMC5801341

[93] Zhou, T. et al. High-content screening in hPSC-neural progenitors identifies drug candidates that inhibit Zika Virus infection in fetal-like organoids and adult brain. *Cell Stem Cell* **21**, 274–283.e5 (2017). 28736217 10.1016/j.stem.2017.06.017 PMC5553280

[94] Pettke, A. et al. Broadly active antiviral compounds disturb Zika virus progeny release rescuing virus-induced toxicity in brain organoids. *Viruses* **13**, 37 (2020). 33383826 10.3390/v13010037 PMC7823652

[95] Huch, M. et al. Long-term culture of genome-stable bipotent stem cells from adult human liver. *Cell* **160**, 299–312 (2015). 25533785 10.1016/j.cell.2014.11.050 PMC4313365

[96] Camp, J. G. et al. Human cerebral organoids recapitulate gene expression programs of fetal neocortex development. *Proc. Natl. Acad. Sci.* **112**, 15672–15677 (2015). 26644564 10.1073/pnas.1520760112 PMC4697386

[97] Hrvatin, S. et al. Differentiated human stem cells resemble fetal, not adult, β cells. *Proc. Natl. Acad. Sci.* **111**, 3038–3043 (2014). 24516164 10.1073/pnas.1400709111 PMC3939927

[98] Nishiga, M., Wang, D. W., Han, Y., Lewis, D. B. & Wu, J. C. COVID-19 and cardiovascular disease: from basic mechanisms to clinical perspectives. *Nat. Rev. Cardiol.* **17**, 543–558 (2020). 32690910 10.1038/s41569-020-0413-9 PMC7370876

[99] Liu, J. et al. Longitudinal characteristics of lymphocyte responses and cytokine profiles in the peripheral blood of SARS-CoV-2 infected patients. *EBioMedicine* **55**, 102763 (2020). 32361250 10.1016/j.ebiom.2020.102763 PMC7165294

[100] Urbischek, M. et al. Organoid culture media formulated with growth factors of defined cellular activity. *Sci. Rep.* **9**, 6193 (2019). 30996238 10.1038/s41598-019-42604-0 PMC6470207

[101] Du, X., Dong, Y., Li, W. & Chen, Y. hPSC-derived lung organoids: potential opportunities and challenges. *Heliyon* **9**, e13498 (2023). 36814627 10.1016/j.heliyon.2023.e13498 PMC9939602

[102] Gard, A. L. et al. High-throughput human primary cell-based airway model for evaluating influenza, coronavirus, or other respiratory viruses in vitro. *Sci. Rep.* **11**, 14961 (2021). 34294757 10.1038/s41598-021-94095-7 PMC8298517

[103] Prioritizing diseases for research and development in emergency contexts. *World Health Organization.* https://www.who.int/activities/prioritizing-diseases-for-research-and-development-in-emergency-contexts*.* (2023).

[104] Hu, B. et al. Characteristics of SARS-CoV-2 and COVID-19. *Nat. Rev. Microbiol.* **19**, 141–154 (2021). 33024307 10.1038/s41579-020-00459-7 PMC7537588

[105] Korteweg, C. & Gu, J. Pathology, molecular biology, and pathogenesis of Avian Influenza A (H5N1) infection in humans. *Am. J. Pathol.* **172**, 1155–1170 (2008). 18403604 10.2353/ajpath.2008.070791 PMC2329826

[106] Martines, R. B., Ng, D. L., Greer, P. W., Rollin, P. E. & Zaki, S. R. Tissue and cellular tropism, pathology and pathogenesis of Ebola and Marburg viruses. *J. Pathol.* **235**, 153–174 (2015). 25297522 10.1002/path.4456

[107] Merad, M. & Martin, J. C. Pathological inflammation in patients with COVID-19: a key role for monocytes and macrophages. *Nat. Rev. Immunol.* **20**, 355–362 (2020). 32376901 10.1038/s41577-020-0331-4 PMC7201395

[108] Lindner, D. et al. Association of cardiac infection with SARS-CoV-2 in confirmed COVID-19 autopsy cases. *JAMA Cardiol.* **5**, 1281 (2020). 32730555 10.1001/jamacardio.2020.3551 PMC7385672

[109] Escher, F. et al. Detection of viral SARS‐CoV‐2 genomes and histopathological changes in endomyocardial biopsies. *ESC Heart Fail.* **7**, 2440–2447 (2020). 32529795 10.1002/ehf2.12805 PMC7307078

[110] Tavazzi, G. et al. Myocardial localization of coronavirus in COVID‐19 cardiogenic shock. *Eur. J. Heart Fail.* **22**, 911–915 (2020). 32275347 10.1002/ejhf.1828 PMC7262276

[111] Yang, L. et al. Cardiomyocytes recruit monocytes upon SARS-CoV-2 infection by secreting CCL2. *Stem Cell Rep.* **16**, 2274–2288 (2021). 10.1016/j.stemcr.2021.07.012 PMC8289700 34403650

[112] Yang, L. et al. An immuno-cardiac model for macrophage-mediated inflammation in COVID-19 hearts. *Circ. Res.* **129**, 33–46 (2021). 33853355 10.1161/CIRCRESAHA.121.319060 PMC8225586

[113] Bhaskar, S. et al. Cytokine Storm in COVID-19—immunopathological mechanisms, clinical considerations, and therapeutic approaches: the REPROGRAM consortium position paper. *Front. Immunol.* **11**, 1648 (2020). 32754159 10.3389/fimmu.2020.01648 PMC7365905

[114] Choi, SS. et al. Organoid modeling of lung-resident immune responses to SARS-CoV-2 infection*.* Preprint at: https://www.researchsquare.com/article/rs-2870695/v1 (2023).

[115] Luukkainen, A. et al. A co-culture Model of PBMC and stem cell derived human nasal epithelium reveals rapid activation of NK and Innate T cells upon influenza a virus infection of the nasal epithelium. *Front. Immunol.* **9**, 2514 (2018). 30467502 10.3389/fimmu.2018.02514 PMC6237251

[116] Lee, J. et al. A multicellular liver organoid model for investigating hepatitis C virus infection and non-alcoholic fatty liver disease progression. *Hepatology* https://journals.lww.com/hep/abstract/9900/a_multicellular_liver_organoid_model_for.655.aspx (2023). 10.1097/HEP.0000000000000683 37976400

[117] Purwada, A. & Singh, A. Immuno-engineered organoids for regulating the kinetics of B-cell development and antibody production. *Nat. Protoc.* **12**, 168–182 (2016). 28005068 10.1038/nprot.2016.157 PMC6355337

[118] Rouse, B. T. & Sehrawat, S. Immunity and immunopathology to viruses: what decides the outcome? *Nat. Rev. Immunol.* **10**, 514–526 (2010). 20577268 10.1038/nri2802 PMC3899649

[119] Sontheimer-Phelps, A., Hassell, B. A. & Ingber, D. E. Modelling cancer in microfluidic human organs-on-chips. *Nat. Rev. Cancer* **19**, 65–81 (2019). 30647431 10.1038/s41568-018-0104-6

[120] van den Berg, A., Mummery, C. L., Passier, R. & van der Meer, A. D. Personalised organs-on-chips: functional testing for precision medicine. *Lab Chip* **19**, 198–205 (2019). 30506070 10.1039/c8lc00827b PMC6336148

[121] Bhatia, S. N. & Ingber, D. E. Microfluidic organs-on-chips. *Nat. Biotechnol.* **32**, 760–772 (2014). 25093883 10.1038/nbt.2989

[122] Balijepalli, A. & Sivaramakrishan, V. Organs-on-chips: research and commercial perspectives. *Drug Discov. Today* **22**, 397–403 (2017). 27866008 10.1016/j.drudis.2016.11.009

[123] Ortega-Prieto, A. M. et al. 3D microfluidic liver cultures as a physiological preclinical tool for hepatitis B virus infection. *Nat. Commun.* **9**, 682 (2018). 29445209 10.1038/s41467-018-02969-8 PMC5813240

[124] Si, L. et al. A human-airway-on-a-chip for the rapid identification of candidate antiviral therapeutics and prophylactics. *Nat. Biomed. Eng.* **5**, 815–829 (2021). 33941899 10.1038/s41551-021-00718-9 PMC8387338

[125] Thacker, V. V. et al. Rapid endotheliitis and vascular damage characterize SARS-CoV-2 infection in a human lung-on-chip model. *EMBO Rep.* **22**, e52744 (2021). 33908688 10.15252/embr.202152744 PMC8183417

[126] Villenave, R. et al. Human Gut-On-A-Chip Supports Polarized Infection of Coxsackie B1 Virus In Vitro. *PLoS One* **12**, e0169412 (2017).. 10.1371/journal.pone.0169412 PMC5287454 28146569

[127] Nawroth, J. C. et al. A microengineered airway lung chip models key features of viral-induced exacerbation of asthma. *Am. J. Respir. Cell Mol. Biol.* **63**, 591–600 (2020). 32706623 10.1165/rcmb.2020-0010MA

[128] Junaid, A. et al. Ebola Hemorrhagic Shock Syndrome-on-a-Chip*. iScience* **23**, 100765 (2020). 10.1016/j.isci.2019.100765 PMC6941864 31887664

[129] Picollet-D’hahan, N., Zuchowska, A., Lemeunier, I. & Le Gac, S. Multiorgan-on-a-Chip: a systemic approach to model and decipher inter-organ communication. *Trends Biotechnol.* **39**, 788–810 (2021). 33541718 10.1016/j.tibtech.2020.11.014

[130] Jeon, J., Choi, N., Lee, S. H. & Sung, J. H. Three-tissue microphysiological system for studying inflammatory responses in gut-liver Axis. *Biomed. Microdevices* **22**, 65 (2020). 32915326 10.1007/s10544-020-00519-y

[131] Adashi, E. Y., O’Mahony, D. P. & Cohen, I. G. The FDA Modernization Act 2.0: drug testing in animals is rendered optional.*Am. J. Med.* **136**, 853–854 (2023). 37080328 10.1016/j.amjmed.2023.03.033

[132] CONGRESS.GOV. H.R. 2617 - Consolidated Appropriations Act, 2023. Became Public Law No: 117-328. December 29, 2022.

